# Dichlorido{2-morpholino-*N*-[1-(2-pyrid­yl)ethyl­idene]ethanamine-κ^3^ *N*,*N*′,*N*′′}zinc(II)

**DOI:** 10.1107/S1600536810050671

**Published:** 2010-12-11

**Authors:** Nurul Azimah Ikmal Hisham, Nura Suleiman Gwaram, Hamid Khaledi, Hapipah Mohd Ali

**Affiliations:** a Department of Chemistry, University of Malaya, 50603 Kuala Lumpur, Malaysia

## Abstract

In the title compound, [ZnCl_2_(C_13_H_19_N_3_O)], the Schiff base ligand acts as an *N*,*N*′,*N*′′-tridentate chelating agent, making two five-membered rings with the Zn^II^ ion. The metal atom is five-coordinated by the Schiff base ligand and two Cl atoms in a distorted square-pyramidal geometry. An intra­molecular C—H⋯Cl inter­action occurs. In the crystal, adjacent mol­ecules are linked together *via* C—H⋯Cl hydrogen-bonding and long range C—H⋯O and C—H⋯Cl inter­actions into a three-dimensional network.

## Related literature

For the crystal structure of an analogous Cd^II^ complex, see: Ikmal Hisham *et al.* (2010 ▶). For crystal structures of similar Zn^II^ complexes, see: Chattopadhyay *et al.* (2009 ▶); Sun (2005 ▶).
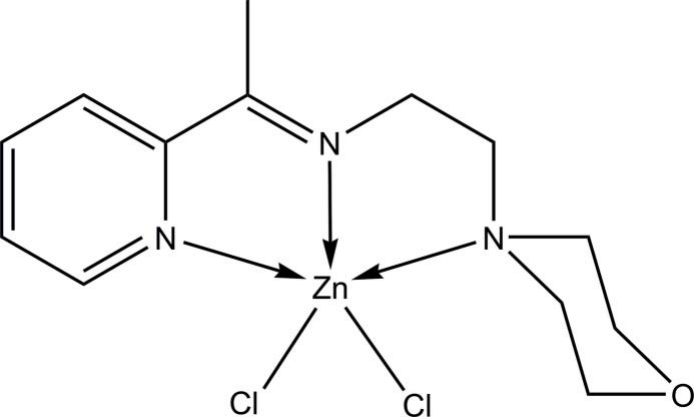


## Experimental

### 

#### Crystal data

[ZnCl_2_(C_13_H_19_N_3_O)]
*M* *_r_* = 369.58
Monoclinic, 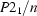

*a* = 9.5737 (12) Å
*b* = 13.7064 (17) Å
*c* = 12.0766 (15) Å
β = 106.643 (2)°
*V* = 1518.3 (3) Å^3^
*Z* = 4
Mo *K*α radiation
μ = 1.97 mm^−1^
*T* = 100 K
0.35 × 0.21 × 0.05 mm

#### Data collection

Bruker APEXII CCD diffractometer
Absorption correction: multi-scan (*SADABS*; Sheldrick, 1996 ▶) *T*_min_ = 0.546, *T*_max_ = 0.908
13693 measured reflections
2979 independent reflections
2552 reflections with *I* > 2σ(*I*)
*R*_int_ = 0.047

#### Refinement

*R*[*F* ^2^ > 2σ(*F* ^2^)] = 0.034
*wR*(*F* ^2^) = 0.091
*S* = 1.08
2979 reflections
182 parameters
H-atom parameters constrained
Δρ_max_ = 1.03 e Å^−3^
Δρ_min_ = −0.39 e Å^−3^

### 

Data collection: *APEX2* (Bruker, 2007 ▶); cell refinement: *SAINT* (Bruker, 2007 ▶); data reduction: *SAINT*; program(s) used to solve structure: *SHELXS97* (Sheldrick, 2008 ▶); program(s) used to refine structure: *SHELXL97* (Sheldrick, 2008 ▶); molecular graphics: *XP* in *SHELXTL* (Sheldrick, 2008 ▶); software used to prepare material for publication: *SHELXL97* and *publCIF* (Westrip, 2010 ▶).

## Supplementary Material

Crystal structure: contains datablocks I, global. DOI: 10.1107/S1600536810050671/pv2366sup1.cif

Structure factors: contains datablocks I. DOI: 10.1107/S1600536810050671/pv2366Isup2.hkl

Additional supplementary materials:  crystallographic information; 3D view; checkCIF report

## Figures and Tables

**Table 1:** Hydrogen-bond geometry (Å, °)

*D*—H⋯*A*	*D*—H	H⋯*A*	*D*⋯*A*	*D*—H⋯*A*
C4—H4⋯Cl1^i^	0.95	2.71	3.625 (3)	161
C7—H7*C*⋯Cl2^ii^	0.98	2.77	3.619 (3)	146
C12—H12*B*⋯Cl1	0.99	2.73	3.526 (3)	138
C10—H10*A*⋯O1^iii^	0.99	2.61	3.408 (3)	137
C9—H9*B*⋯Cl1^iv^	0.99	2.88	3.665 (3)	137
C7—H7*B*⋯Cl1^i^	0.98	2.85	3.807 (3)	166
C8—H8*A*⋯Cl2^iv^	0.99	2.87	3.750 (3)	149

Symmetry codes: (i) 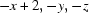; (ii) 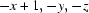; (iii) 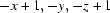; (iv) 
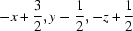
.

**Fig. 1.**
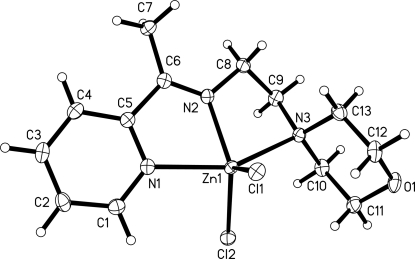
Displacement ellipsoid plot of the title compound at the 50% probability level. G atoms are drawn as spheres of arbitrary radius.

## supplementary crystallographic information

### Comment

The title compound is isostructural with the analogous Cd^II^ complex (Ikmal Hisham *et al.*, 2010). The Schiff base, 2-morpholino-*N*-[1-(2-pyridyl)ethylidene]ethanamine and two Cl atoms coordinate the Zn^II^ ion in a distorted square-pyramidal geometry (τ = 0.22). The Zn—Cl and Zn—N bond lengths in the structure are in agreement with the values reported in the literature (Chattopadhyay *et al.*, 2009; Sun, 2005). In the crystal structure, intermolecular C—H···Cl hydrogen bonding and long range C—H···O and C—H···Cl interactions link the adjacent molecules into a three-dimensional network. An intramolecular C—H···Cl hydrogen bonding has also been observed.

### Experimental

A mixture of 2-acetylpyridine (0.20 g, 1.65 mmol) and 4-(2-aminoethyl)morpholine (0.21 g, 1.65 mmol) in the presence of a few drops of HCl (37%) in ethanol (20 ml) was refluxed. After 2 hr a solution of zinc(II) acetate dihydrate (0.36 g, 1.65 mmol) in a minimum amount of water was added and the resulting solution was refluxed for 30 min, then set aside at room temperature. The crystals of the title complex were obtained after a few days.

### Refinement

The hydrogen atoms were placed at calculated positions (C—H 0.95 - 0.99 Å) and were treated as riding on their parent atoms with *U*(H) set to 1.2–1.5 *Ueq*(C). The final difference map was essentially featurless with residual electron density close to the metal atom.

### Crystal data

**Table:**

[ZnCl_2_(C_13_H_19_N_3_O)]	*F*(000) = 760
*M**_r_* = 369.58	*D*_x_ = 1.617 Mg m^−^^3^
Monoclinic, *P*2_1_/*n*	Mo *K*α radiation, λ = 0.71073 Å
Hall symbol: -P 2yn	Cell parameters from 3471 reflections
*a* = 9.5737 (12) Å	θ = 2.3–27.5°
*b* = 13.7064 (17) Å	µ = 1.97 mm^−^^1^
*c* = 12.0766 (15) Å	*T* = 100 K
β = 106.643 (2)°	Plate, yellow
*V* = 1518.3 (3) Å^3^	0.35 × 0.21 × 0.05 mm
*Z* = 4	

### Data collection

**Table:**

Bruker APEXII CCD diffractometer	2979 independent reflections
Radiation source: fine-focus sealed tube	2552 reflections with *I* > 2σ(*I*)
graphite	*R*_int_ = 0.047
φ and ω scans	θ_max_ = 26.0°, θ_min_ = 2.3°
Absorption correction: multi-scan (*SADABS*; Sheldrick, 1996)	*h* = −11→11
*T*_min_ = 0.546, *T*_max_ = 0.908	*k* = −16→16
13693 measured reflections	*l* = −14→14

### Refinement

**Table:**

Refinement on *F*^2^	Primary atom site location: structure-invariant direct methods
Least-squares matrix: full	Secondary atom site location: difference Fourier map
*R*[*F*^2^ > 2σ(*F*^2^)] = 0.034	Hydrogen site location: inferred from neighbouring sites
*wR*(*F*^2^) = 0.091	H-atom parameters constrained
*S* = 1.08	*w* = 1/[σ^2^(*F*_o_^2^) + (0.0515*P*)^2^ + 0.4492*P*] where *P* = (*F*_o_^2^ + 2*F*_c_^2^)/3
2979 reflections	(Δ/σ)_max_ = 0.001
182 parameters	Δρ_max_ = 1.03 e Å^−^^3^
0 restraints	Δρ_min_ = −0.39 e Å^−^^3^

### Special details

**Table:**

Geometry. All e.s.d.'s (except the e.s.d. in the dihedral angle between two l.s. planes) are estimated using the full covariance matrix. The cell e.s.d.'s are taken into account individually in the estimation of e.s.d.'s in distances, angles and torsion angles; correlations between e.s.d.'s in cell parameters are only used when they are defined by crystal symmetry. An approximate (isotropic) treatment of cell e.s.d.'s is used for estimating e.s.d.'s involving l.s. planes.
Refinement. Refinement of *F*^2^ against ALL reflections. The weighted *R*-factor *wR* and goodness of fit *S* are based on *F*^2^, conventional *R*-factors *R* are based on *F*, with *F* set to zero for negative *F*^2^. The threshold expression of *F*^2^ > σ(*F*^2^) is used only for calculating *R*-factors(gt) *etc*. and is not relevant to the choice of reflections for refinement. *R*-factors based on *F*^2^ are statistically about twice as large as those based on *F*, and *R*- factors based on ALL data will be even larger.

### Fractional atomic coordinates and isotropic or equivalent isotropic displacement parameters (Å^2^)

**Table:**

	*x*	*y*	*z*	*U*_iso_*/*U*_eq_	
Zn1	0.72733 (3)	0.08854 (2)	0.16005 (3)	0.01368 (12)	
Cl1	0.95230 (7)	0.13655 (5)	0.26836 (6)	0.01677 (16)	
Cl2	0.54542 (7)	0.19943 (5)	0.13205 (6)	0.01828 (17)	
O1	0.6851 (2)	0.06197 (15)	0.51473 (16)	0.0224 (4)	
N1	0.7630 (2)	0.11923 (16)	−0.01195 (19)	0.0156 (5)	
N2	0.7468 (2)	−0.04614 (15)	0.08710 (18)	0.0145 (5)	
N3	0.6560 (2)	−0.01499 (15)	0.28543 (18)	0.0146 (5)	
C1	0.7536 (3)	0.20493 (19)	−0.0655 (2)	0.0183 (6)	
H1	0.7219	0.2601	−0.0317	0.022*	
C2	0.7881 (3)	0.2169 (2)	−0.1689 (2)	0.0208 (6)	
H2	0.7788	0.2788	−0.2057	0.025*	
C3	0.8363 (3)	0.1366 (2)	−0.2170 (2)	0.0191 (6)	
H3	0.8633	0.1430	−0.2865	0.023*	
C4	0.8449 (3)	0.0468 (2)	−0.1625 (2)	0.0172 (6)	
H4	0.8764	−0.0095	−0.1946	0.021*	
C5	0.8064 (3)	0.04073 (18)	−0.0604 (2)	0.0147 (6)	
C6	0.7974 (3)	−0.05297 (19)	0.0006 (2)	0.0148 (6)	
C7	0.8414 (3)	−0.14742 (19)	−0.0420 (2)	0.0178 (6)	
H7A	0.8954	−0.1870	0.0240	0.027*	
H7B	0.9034	−0.1342	−0.0922	0.027*	
H7C	0.7541	−0.1829	−0.0856	0.027*	
C8	0.7172 (3)	−0.13162 (19)	0.1493 (2)	0.0186 (6)	
H8A	0.8078	−0.1542	0.2064	0.022*	
H8B	0.6782	−0.1856	0.0947	0.022*	
C9	0.6064 (3)	−0.10075 (19)	0.2097 (2)	0.0184 (6)	
H9A	0.5134	−0.0847	0.1511	0.022*	
H9B	0.5879	−0.1558	0.2567	0.022*	
C10	0.5308 (3)	0.0196 (2)	0.3241 (2)	0.0181 (6)	
H10A	0.4885	−0.0360	0.3557	0.022*	
H10B	0.4546	0.0459	0.2570	0.022*	
C11	0.5765 (3)	0.0979 (2)	0.4155 (2)	0.0217 (6)	
H11A	0.6157	0.1546	0.3833	0.026*	
H11B	0.4905	0.1200	0.4386	0.026*	
C12	0.8104 (3)	0.0335 (2)	0.4810 (2)	0.0208 (6)	
H12A	0.8865	0.0092	0.5495	0.025*	
H12B	0.8501	0.0910	0.4507	0.025*	
C13	0.7736 (3)	−0.0452 (2)	0.3896 (2)	0.0197 (6)	
H13A	0.8621	−0.0616	0.3666	0.024*	
H13B	0.7425	−0.1046	0.4226	0.024*	

### Atomic displacement parameters (Å^2^)

**Table:**

	*U*^11^	*U*^22^	*U*^33^	*U*^12^	*U*^13^	*U*^23^
Zn1	0.01259 (19)	0.01472 (18)	0.01459 (18)	−0.00019 (11)	0.00529 (13)	−0.00150 (11)
Cl1	0.0128 (3)	0.0199 (3)	0.0179 (3)	−0.0021 (2)	0.0048 (3)	−0.0023 (2)
Cl2	0.0152 (3)	0.0206 (3)	0.0194 (3)	0.0037 (3)	0.0056 (3)	0.0007 (2)
O1	0.0203 (11)	0.0347 (11)	0.0144 (10)	0.0023 (9)	0.0085 (9)	0.0005 (8)
N1	0.0126 (12)	0.0185 (11)	0.0154 (11)	−0.0004 (9)	0.0037 (9)	0.0002 (9)
N2	0.0138 (12)	0.0159 (11)	0.0133 (11)	−0.0016 (9)	0.0030 (9)	−0.0005 (9)
N3	0.0142 (12)	0.0156 (11)	0.0145 (11)	−0.0015 (9)	0.0051 (9)	−0.0004 (9)
C1	0.0172 (14)	0.0179 (13)	0.0186 (14)	−0.0009 (11)	0.0033 (12)	−0.0023 (10)
C2	0.0228 (16)	0.0228 (14)	0.0146 (13)	−0.0042 (12)	0.0019 (12)	0.0021 (10)
C3	0.0148 (14)	0.0292 (15)	0.0135 (13)	−0.0032 (12)	0.0044 (11)	−0.0010 (11)
C4	0.0108 (14)	0.0222 (14)	0.0186 (14)	−0.0002 (11)	0.0044 (11)	−0.0034 (11)
C5	0.0119 (13)	0.0168 (13)	0.0137 (13)	−0.0013 (10)	0.0010 (11)	−0.0018 (10)
C6	0.0099 (13)	0.0187 (13)	0.0139 (13)	−0.0015 (10)	0.0005 (11)	−0.0010 (10)
C7	0.0171 (14)	0.0179 (13)	0.0201 (14)	−0.0003 (11)	0.0081 (12)	−0.0026 (10)
C8	0.0248 (16)	0.0147 (13)	0.0174 (14)	−0.0013 (11)	0.0077 (12)	0.0003 (10)
C9	0.0171 (15)	0.0190 (13)	0.0203 (14)	−0.0056 (11)	0.0070 (12)	−0.0013 (11)
C10	0.0110 (14)	0.0257 (15)	0.0190 (14)	−0.0028 (11)	0.0065 (11)	0.0018 (11)
C11	0.0212 (16)	0.0268 (15)	0.0196 (15)	0.0055 (12)	0.0099 (13)	0.0007 (11)
C12	0.0113 (14)	0.0355 (16)	0.0149 (13)	−0.0003 (12)	0.0028 (11)	0.0017 (12)
C13	0.0166 (15)	0.0231 (14)	0.0194 (14)	0.0026 (11)	0.0052 (12)	0.0039 (11)

### Geometric parameters (Å, °)

**Table:**

Zn1—N2	2.077 (2)	C4—H4	0.9500
Zn1—N1	2.239 (2)	C5—C6	1.495 (4)
Zn1—Cl2	2.2628 (7)	C6—C7	1.497 (4)
Zn1—Cl1	2.2736 (7)	C7—H7A	0.9800
Zn1—N3	2.316 (2)	C7—H7B	0.9800
O1—C12	1.427 (3)	C7—H7C	0.9800
O1—C11	1.430 (3)	C8—C9	1.510 (4)
N1—C1	1.331 (3)	C8—H8A	0.9900
N1—C5	1.346 (3)	C8—H8B	0.9900
N2—C6	1.274 (3)	C9—H9A	0.9900
N2—C8	1.463 (3)	C9—H9B	0.9900
N3—C9	1.482 (3)	C10—C11	1.511 (4)
N3—C10	1.484 (3)	C10—H10A	0.9900
N3—C13	1.487 (3)	C10—H10B	0.9900
C1—C2	1.390 (4)	C11—H11A	0.9900
C1—H1	0.9500	C11—H11B	0.9900
C2—C3	1.384 (4)	C12—C13	1.511 (4)
C2—H2	0.9500	C12—H12A	0.9900
C3—C4	1.387 (4)	C12—H12B	0.9900
C3—H3	0.9500	C13—H13A	0.9900
C4—C5	1.387 (4)	C13—H13B	0.9900
			
N2—Zn1—N1	73.58 (8)	C6—C7—H7B	109.5
N2—Zn1—Cl2	133.83 (6)	H7A—C7—H7B	109.5
N1—Zn1—Cl2	92.90 (6)	C6—C7—H7C	109.5
N2—Zn1—Cl1	108.41 (6)	H7A—C7—H7C	109.5
N1—Zn1—Cl1	96.22 (6)	H7B—C7—H7C	109.5
Cl2—Zn1—Cl1	116.95 (3)	N2—C8—C9	106.9 (2)
N2—Zn1—N3	79.16 (8)	N2—C8—H8A	110.4
N1—Zn1—N3	150.85 (8)	C9—C8—H8A	110.4
Cl2—Zn1—N3	98.68 (6)	N2—C8—H8B	110.4
Cl1—Zn1—N3	102.08 (6)	C9—C8—H8B	110.4
C12—O1—C11	108.9 (2)	H8A—C8—H8B	108.6
C1—N1—C5	118.7 (2)	N3—C9—C8	112.0 (2)
C1—N1—Zn1	127.53 (19)	N3—C9—H9A	109.2
C5—N1—Zn1	113.63 (17)	C8—C9—H9A	109.2
C6—N2—C8	122.5 (2)	N3—C9—H9B	109.2
C6—N2—Zn1	120.96 (18)	C8—C9—H9B	109.2
C8—N2—Zn1	116.09 (17)	H9A—C9—H9B	107.9
C9—N3—C10	107.6 (2)	N3—C10—C11	111.5 (2)
C9—N3—C13	109.5 (2)	N3—C10—H10A	109.3
C10—N3—C13	107.9 (2)	C11—C10—H10A	109.3
C9—N3—Zn1	100.66 (15)	N3—C10—H10B	109.3
C10—N3—Zn1	115.21 (16)	C11—C10—H10B	109.3
C13—N3—Zn1	115.49 (16)	H10A—C10—H10B	108.0
N1—C1—C2	122.7 (3)	O1—C11—C10	110.9 (2)
N1—C1—H1	118.7	O1—C11—H11A	109.5
C2—C1—H1	118.7	C10—C11—H11A	109.5
C3—C2—C1	118.5 (3)	O1—C11—H11B	109.5
C3—C2—H2	120.8	C10—C11—H11B	109.5
C1—C2—H2	120.8	H11A—C11—H11B	108.1
C2—C3—C4	119.2 (3)	O1—C12—C13	111.4 (2)
C2—C3—H3	120.4	O1—C12—H12A	109.4
C4—C3—H3	120.4	C13—C12—H12A	109.4
C3—C4—C5	118.7 (3)	O1—C12—H12B	109.4
C3—C4—H4	120.6	C13—C12—H12B	109.4
C5—C4—H4	120.6	H12A—C12—H12B	108.0
N1—C5—C4	122.1 (2)	N3—C13—C12	112.4 (2)
N1—C5—C6	113.8 (2)	N3—C13—H13A	109.1
C4—C5—C6	123.9 (2)	C12—C13—H13A	109.1
N2—C6—C5	115.5 (2)	N3—C13—H13B	109.1
N2—C6—C7	123.8 (2)	C12—C13—H13B	109.1
C5—C6—C7	120.8 (2)	H13A—C13—H13B	107.9
C6—C7—H7A	109.5		

### Hydrogen-bond geometry (Å, °)

**Table:**

*D*—H···*A*	*D*—H	H···*A*	*D*···*A*	*D*—H···*A*
C4—H4···Cl1^i^	0.95	2.71	3.625 (3)	161
C7—H7C···Cl2^ii^	0.98	2.77	3.619 (3)	146
C12—H12B···Cl1	0.99	2.73	3.526 (3)	138
C10—H10A···O1^iii^	0.99	2.61	3.408 (3)	137
C9—H9B···Cl1^iv^	0.99	2.88	3.665 (3)	137
C7—H7B···Cl1^i^	0.98	2.85	3.807 (3)	166
C8—H8A···Cl2^iv^	0.99	2.87	3.750 (3)	149

Symmetry codes: (i) −*x*+2, −*y*, −*z*; (ii) −*x*+1, −*y*, −*z*; (iii) −*x*+1, −*y*, −*z*+1; (iv) −*x*+3/2, *y*−1/2, −*z*+1/2.
